# Cardio-facio-cutaneous syndrome and gastrointestinal defects: report on a newborn with 19p13.3 deletion including the *MAP 2 K2* gene

**DOI:** 10.1186/s13052-022-01241-6

**Published:** 2022-05-04

**Authors:** Gregorio Serra, Sofia Felice, Vincenzo Antona, Maria Rita Di Pace, Mario Giuffrè, Ettore Piro, Giovanni Corsello

**Affiliations:** Department of Health Promotion, Mother and Child Care, Internal Medicine and Medical Specialties “G. D’Alessandro”, University of Palermo, Palermo, Italy

**Keywords:** CFCS, RASopathies, Contiguous gene syndrome, Array-CGH, Genotype-phenotype correlations, HPS, Case report

## Abstract

**Background:**

Cardio-facio-cutaneous syndrome (CFCS) belongs to RASopathies, a group of conditions caused by mutations in genes encoding proteins of the rat sarcoma/mitogen-activated protein kinase (RAS/MAPK) pathway. It is a rare syndrome, with about 300 patients reported. Main clinical manifestations include facial dysmorphisms, growth failure, heart defects, developmental delay, and ectodermal abnormalities. Mutations (mainly missense) of four genes (*BRAF*, *MAP 2 K1*, *MAP 2 K2*, and *KRAS*) have been associated to CFCS. However, whole gene deletions/duplications and chromosomal microdeletions have been also reported. Specifically, 19p13.3 deletion including *MAP 2 K2* gene are responsible for cardio-facio-cutaneous microdeletion syndrome, whose affected subjects show more severe phenotype than CFCS general population.

**Case presentation:**

Hereby, we report on a female newborn with prenatal diagnosis of omphalocele, leading to further genetic investigations through amniocentesis. Among these, array comparative genomic hybridization (a-CGH) identified a 19p13.3 microdeletion, spanning 1.27 Mb and including *MAP 2 K2* gene. Clinical features at birth (coarse face with dysmorphic features, sparse and friable hair, cutaneous vascular malformations and hyperkeratotic lesions, interventricular septal defect, and omphalocele) were compatible with CFCS diagnosis, and further postnatal genetic investigations were not considered necessary. Soon after discharge, at around 1 month of life, she was readmitted to our Neonatal Intensive Care Unit due to repeated episodes of vomiting, subtending a hypertrophic pyloric stenosis (HPS) which was promptly identified and treated.

**Conclusions:**

Our report supports the 19p13.3 microdeletion as a contiguous gene syndrome, in which the involvement of the genes contiguous to *MAP 2 K2* may modify the patients’ phenotype. It highlights how CFCS affected subjects, including those with 19p13.3 deletions, may have associated gastrointestinal defects (e.g., omphalocele and HPS), providing further data on 19p13.3 microdeletion syndrome, and a better characterization of its genomic and phenotypic features. The complex clinical picture of such patients may be worsened by additional, and even precocious, life-threatening conditions like HPS. Clinicians must consider, anticipate and/or promptly treat possible medical and surgical complications, with the aim of reducing adverse outcomes. Extensive diagnostic work-up, and early, continuous, and multidisciplinary follow-up, as well as integrated care, are necessary for the longitudinal clinical evolution of any single patient.

## Background

Cardio-facio-cutaneous syndrome (CFCS) belongs to RASopathies, a group of conditions caused by germline mutations in genes encoding components or regulators of the rat sarcoma/mitogen-activated protein kinase (RAS/MAPK) pathway [1], main mechanism regulating cell growth and differentiation, proliferation, migration, and apoptosis [2]. It is a rare condition, with about 300 reported patients. Main clinical manifestations include facial dysmorphisms, growth failure, heart defects, developmental delay including intellectual disability, and ectodermal abnormalities (sparse and friable hair, hyperkeratotic and/or generalized ichthyosis-like lesions). Mutations of four genes have been associated to CFCS: *BRAF* (7q34), *MAP 2 K1* (15q22.31), *MAP 2 K2* (19p13.3, Cardio-facio-cutaneous syndrome 4, OMIM #615280), and *KRAS* (12p12.1). They encode for the RAS protein (KRAS) and/or its downstream signaling serine-threonine kinases (BRAF, MEK1, MEK2). Most of them are missense variants. However, whole gene deletions or duplications and chromosomal microdeletions have been also reported. Chromosome region 19p13.3 harbors *MAP 2 K2* gene. Deletions of such genomic band including this gene are associated to cardio-facio-cutaneous microdeletion syndrome, whose affected subjects show more severe phenotype than CFCS general population.

Hereby, we report on a female newborn with prenatal diagnosis of omphalocele, leading to further genetic investigations through amniocentesis. Among these, array comparative genomic hybridization (CGH) identified a 19p13.3 deletion, including *MAP 2 K2* gene. Clinical features at birth were compatible with CFCS diagnosis, and further postnatal genetic investigations were not considered necessary. Soon after discharge, at around 1 month of life, she was readmitted to our Neonatal Intensive Care Unit (NICU), due to repeated episodes of vomiting. A hypertrophic pyloric stenosis was, then, also found and promptly treated.

## Case presentation

**Fig. 1:**
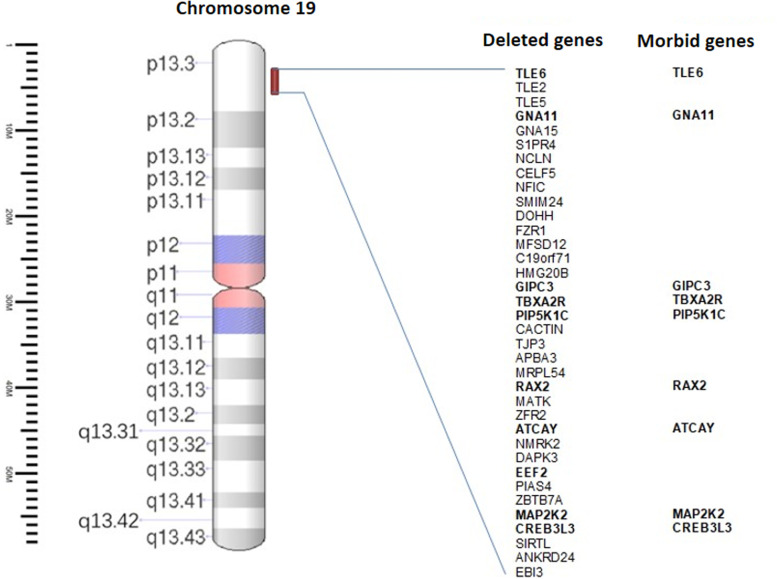
Overview of the 19p13.3 region and its gene content, showing present patient’s microdeletion spanning about 1.27 Mb of genomic DNA, from position 2,980,128 to 4,251,077, according to DECIPHER Genome Browser (GRCh37/hg19 assembly) [3]

**Fig. 2:**
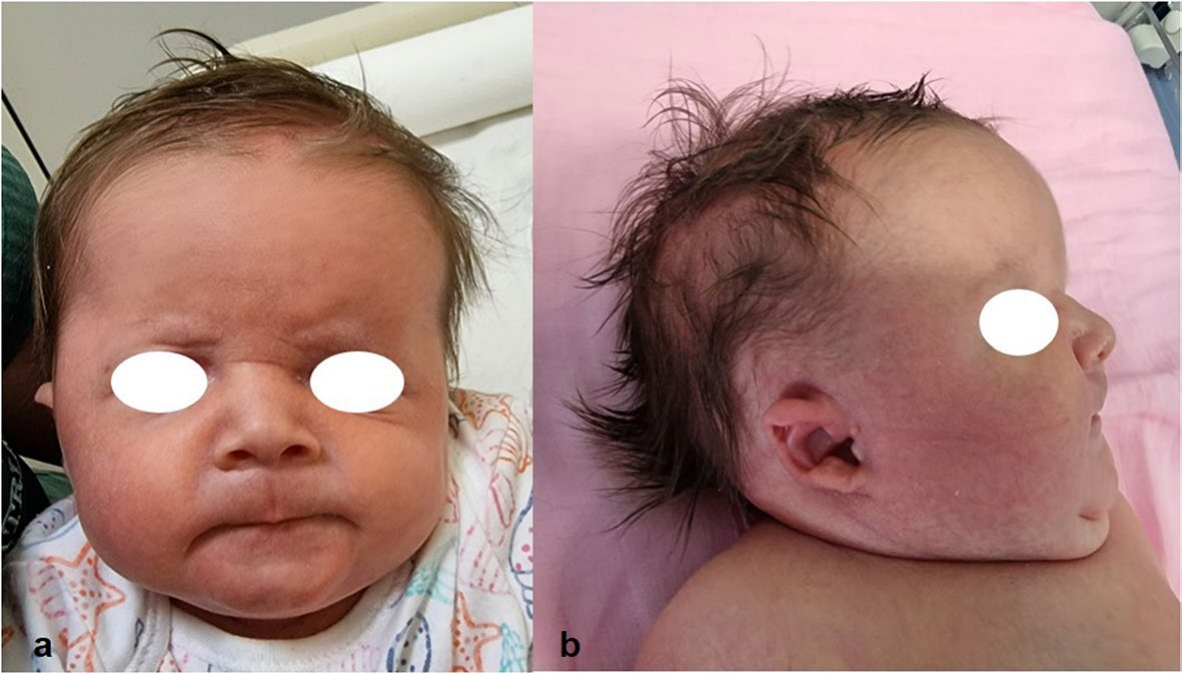
**a**. Patient’s front view: coarse face and asymmetry due to left hypoplasia, high and prominent forehead, hypoplastic supraorbital ridges, sparse and friable hair, eyebrows and eyelashes, wide and depressed nasal bridge, bulbous tip, anteverted nares, long philtrum; **b**. Lateral view: bilateral small, dysplastic, crumbled and posteriorly rotated ears with thickened helices and right preauricular tag

**Fig. 3:**
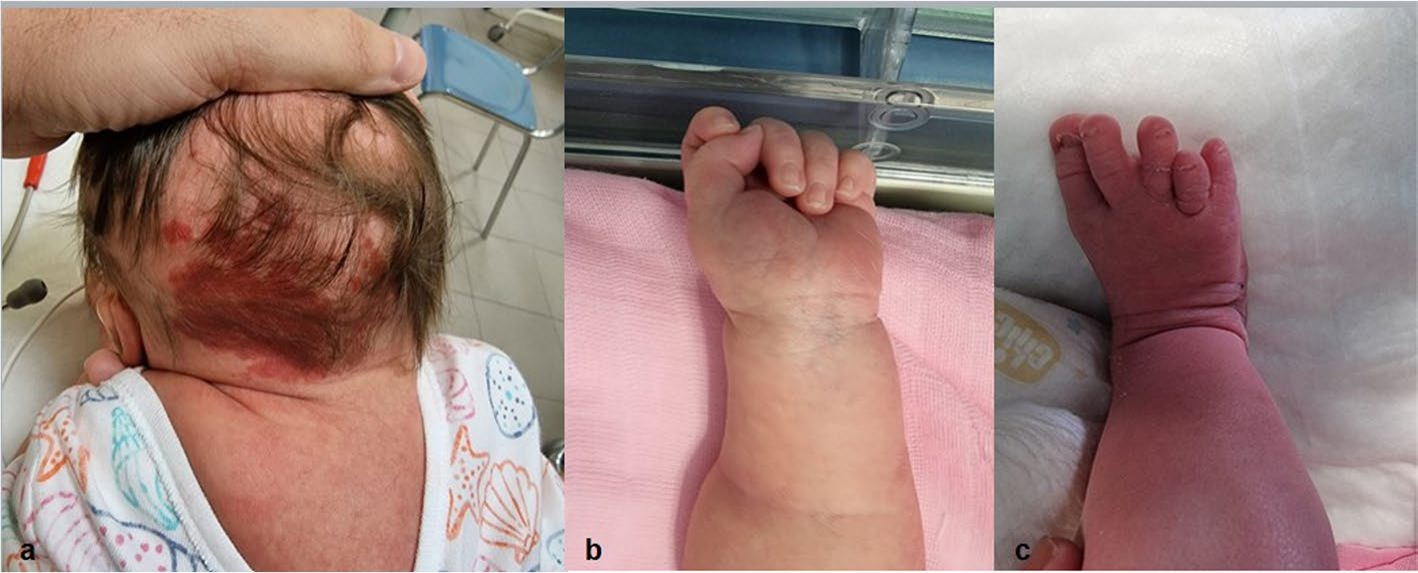
**a**. Wide cutaneous vascular malformations posteriorly in the neck and occiput; **b**. Adducted thumb; **c**. Syndactyly of the right 2nd and 3rd toes, broad 1st, proximal set of the 4th, and clinodactyly of the 5th ones

A female newborn was the first child of healthy and nonconsanguineous parents. Family history was unremarkable. Pregnancy was marked by gestational diabetes, treated with insulin. The first prenatal ultrasound (US), performed at 14 weeks of gestation (WG), disclosed omphalocele. Then, further genetic investigations through amniocentesis were recommended and performed. Karyotype analysis showed a normal female chromosomal set, and molecular analysis of 11p15 genomic region did not disclose abnormalities. Conversely, a-CGH (100–150 Kb resolution, genome assembly GRCh37.p13) identified a 19p13.3 deletion. The genomic rearrangement of 1.27 Mb spanned from position 2,980,128 to 4,251,077, and included, besides *MAP 2 K2*, further genes: *TLE6*, *GNA11*, *GIPC3*, *TBXA2R*, *PIP5K1C*, *RAX2*, *ATCAY*, *EEF2* and *CREB3L3* (Fig. 1). The following prenatal US evaluations documented increased head circumference (+ 2 standard deviation, SD) in addition to polyhydramnios, but ruled out other major malformations. A female newborn was delivered at 38 WG by caesarean section. At birth, anthropometric measurements were as follows: weight 4040 g (>99th centile, + 2.82 SD), length 50 cm (81st centile, + 0.88 SD), and occipitofrontal circumference (OFC) 36 cm (99th centile, + 2.23 SD). Apgar scores were 7, 8, and 9 at 1, 5 and 10 min respectively. Postnatally, due to respiratory distress which needed non-invasive ventilatory support (continuous positive airway pressure administration), she was transferred to our NICU. At admission, physical examination showed macrocephaly, high and prominent forehead, hypoplastic supraorbital ridges, facial asymmetry due to left hypoplasia, coarse face, sparse and friable hair, eyebrows, and eyelashes, left palpebral ptosis, down slanting palpebral fissures, wide and depressed nasal bridge, bulbous tip, anteverted nares, long philtrum and macroglossia. Bilateral small, dysplastic, crumbled and posteriorly rotated ears, with thickened helices and right preauricular tag completed her craniofacial profile (Fig. 2a/b). Wide cutaneous vascular malformations posteriorly in the neck and occiput, hyperkeratotic lesions in the right eyebrow skin region, bilateral adducted thumb, syndactyly of the right 2nd and 3rd toes, broad 1st, proximal set of the 4th and clinodactyly of the 5th ones, as well as omphalocele were also observed (Fig. 3a/b/c). Mild generalized hypotonia, in addition to poor reactivity, spontaneous motor activity and suction outlined her neurological profile. Laboratory examinations, including complete blood count, serum electrolytes, liver and kidney function tests, showed normal results. Head US revealed hypoplasia of cerebellum and body and *splenium* of the corpus callosum, as well as increased size of the lateral ventricles. Heart US identified two mid-apical muscular interventricular septal defects, in addition to mild supravalvular aortic dilation, bovine aortic arch, and patent *foramen ovale*. Ophthalmological evaluation, and hearing screening through transient evoked otoacoustic emissions (TEOAEs), revealed no abnormalities.

In the second day of life, our baby underwent surgery to repair omphalocele. The following postoperative clinical evolution was characterized by initial feeding difficulties, which required nasogastric tube support, and subsequent gradual recovery with achievement of optimal exclusive breastfeeding at around 2 weeks of life. In the meantime, she briefly (for about 24 h after the surgical correction) needed invasive mechanical ventilation, and then exclusively oxygen support until the third week of life. Then, the clinical course occurred without complications, and she was discharged in good general conditions and adequate weight and length growth at age 1 month. After 6 days, the baby presented with recurrent vomiting. Thus, she was readmitted to our NICU, where an abdominal US was soon carried out. This showed pyloric muscle thickness (MT) of 4–5 mm, and channel length (CL) 18 mm, according with a hypertrophic pyloric stenosis (HPS) diagnosis. She then begun intravenous rehydration and whole parenteral nutrition, and underwent Ramstedt pyloromyotomy 3 days later. Feeding through nasogastric tube was started on day 3 after surgery, and enteral nutrition was gradually increased. The patient was discharged 1 week after the surgical operation, tolerating full oral feeding. She had a normal clinical evolution, with adequate weight gain, and is included in a multidisciplinary follow-up. She currently is 2 months and 24 days old, and her anthropometric measures, according to World Health Organization growth chart for neonatal and infant close monitoring [4], are: weight 4960 g (14th centile, − 1.10 SD), length 58 cm (27th centile, -0.60 SD) and OFC 39 cm (41th centile, − 0.22 SD). She shows mild developmental delay, with axial central type hypotonia, normal tone of the limbs and archaic and osteotendinous reflexes. She has no other clinical and/or instrumental abnormalities, except for a plane vascular malformation in the dorsal region (maximum diameters 0.7 × 0.5 cm).

## Discussion and conclusions

Cardio-facio-cutaneous syndrome (CFCS) is a congenital disorder characterized by distinctive craniofacial features, heart defects (CHD, including pulmonic stenosis, atrial septal defect, and hypertrophic cardiomyopathy), developmental delay/intellectual disability, and ectodermal abnormalities (sparse and friable hair, hyperkeratotic skin lesions, generalized ichthyosis-like conditions). Typical facial dysmorphisms are high forehead with bitemporal narrowing, hypoplastic supraorbital ridges, down slanting palpebral fissures, depressed nasal bridge, and posteriorly angulated ears with prominent helices [5]. Craniofacial features are like those observed in Noonan syndrome. However, CFCS patients usually have more severe medical complications and developmental delay/intellectual disability [6]. Then, CFCS clinical features overlap with those of Noonan and Costello syndromes, but skin involvement is specific for CFCS, allowing to distinguish it from other RASopathies. Indeed, all CFCS patients develop some form of cutaneous lesions and, therefore, dermatological consultation and follow-up are recommended. Specifically, infantile hemangiomas are seen in 25% of cases (higher than other RASopathies) [7] and were observed also in the present patient.

Chromosome 19 has the highest gene density within human DNA. However, deletions and duplications of the 19p13.3 terminal band are poorly reported [8]. The microdeletion found in our patient partially matches other deletions, within the same region, previously described in patients showing variable CFCS phenotype (mostly overlapping to that of the proband). This further supports the evidence of a recognizable clinical picture [9, 10], although in none of the reported affected subjects’ gastrointestinal defects, like those seen in our patient, were observed [11]. Gastrointestinal anomalies have been frequently observed in CFCS patients [12], but omphalocele and HPS are scarcely documented.

Activating gain-of-function missense mutations of *MAP 2 K2* are mostly responsible for CFCS. However, although rarely reported, the syndrome is also associated to *MAP 2 K2* haploinsufficiency, or to 19p13.3 microdeletion including such gene. The 1.27 Mb rearrangement identified in our patient contains, besides *MAP 2 K2*, about thirty further genes, and nine of them are known to be disease causing (*TLE6*, *GNA11*, *GIPC3*, *TBXA2R*, *PIP5K1C*, *RAX2*, *ATCAY*, *EEF2* and *CREB3L3*) (Fig. 3). *GIPC3* encodes for a protein, highly expressed in the spinal ganglion and inner ear sensitive hairy cells. Its mutations cause an autosomal recessive form of non-syndromic sensorineural deafness [13]. It has, moreover, a high expression in small intestine and jejunum, and then its deletions may contribute to the gastrointestinal defects observed in our patient. *TBXA2R* mutations are associated to platelet-related bleeding [14]. *PIP5K1C* mutations cause autosomal recessive lethal congenital contractural syndrome type 3 [15]. *RAX2* is expressed in the retina, and its mutations are associated to macular degeneration and cone rod dystrophy [16]. *ATCAY* mutations may cause autosomal recessive Cayman cerebellar ataxia, while those of *EEF2* (MIM #130610) spinocerebellar ataxia [17]. Finally, heterozygous variants of *CREB3L3* (MIM #661998) are associated to hypertriglyceridemia [18]. Our patient shows cerebellar hypoplasia and mild developmental delay, but no hematological (platelet alterations), metabolic (lipidic profile), and ocular abnormalities, although the appearance of such anomalies over time cannot be ruled out. The other genes in the deleted region may also play a causative role for the phenotype, but it is hard to establish their involvement as well as genotype-phenotype correlations [19, 20]. However, our results support the evidence of 19p13.3 microdeletion as contiguous gene syndrome, in which some of the genes contiguous to *MAP 2 K2* may modify patients’ phenotype (e.g. *GIPC3* associated to gastrointestinal defects) [21, 22].

Our report highlights how CFCS patients, including those with 19p13.3 deletions, may have associated gastrointestinal defects (e.g. omphalocele and HPS). It provides further data on 19p13.3 microdeletion syndrome expanding the phenotype, in view of a better characterization of its genomic and clinical features. More patients with overlapping deletions are needed to support our findings, and to define the contribution of the involved genes.

Extensive diagnostic work-up, as well as early, continuous, long-term and multidisciplinary follow-up [23] (oncologic for increased risk of acute lymphoblastic leukemia, non-Hodgkin lymphoma, Langerhans cell histiocytosis, and hepatoblastoma [24, 25]; cardiologic; neurodevelopmental; dermatological, also for vascular malformations and angiomas; ophthalmological/audiological, and surgical/gastroenterological for risk of HPS, umbilical hernia, anal atresia and other malformations) and integrated care, are necessary for these patients. Their complex clinical phenotype may be worsened by additional, and even early, life-threatening conditions like HPS, as occurred in the present patient [26, 27]. Clinicians must consider and promptly treat the possible medical and surgical complications, with the aim of reducing the adverse outcomes according to an individualized approach.

## Data Availability

The datasets used and analyzed during the current study are available from the corresponding author on reasonable request.

## Abbreviations

aCGH: Array comparative genomic hybridization
CHD: Congenital heart defect
CL: Channel length
FISH: Fluorescence in situ hybridization
HPS: Hypertrophic pyloric stenosis
MT: Muscle thickness
NICU: Neonatal intensive care unit
OFC: Occipitofrontal circumference
SD: Standard deviation
TEOAEs: Transient evoked otoacoustic emissions
US: Ultrasonography
WG: Weeks of gestation

## References

[1] Gos M, Smigiel R, Kaczan T, Landowska A, Abramowicz A, Sasiadek M, Bal J (2018). MAP 2K2 mutation as a cause of cardio-facio-cutaneous syndrome in an infant with a severe and fatal course of the disease. Am J Med Genet A.

[2] Bezniakow N, Gos M, Obersztyn E (2014). The RASopathies as an example of RAS/MAPK pathway disturbances - clinical presentation and molecular pathogenesis of selected syndromes. Dev Period Med.

[3] DECIPHER (DatabasE of genomiC varIation and Phenotype in Humans using Ensembl Resources). https://www.deciphergenomics.org/browser#q/19p13.3/location/19:1-7025000. 10.1016/j.ajhg.2009.03.010 PMC2667985 19344873

[4] World Health Organization. Child growth standards 2021. https://www.who.int/tools/child-growth-standards/standards.

[5] Pierpont ME, Magoulas PL, Adi S, Kavamura MI, Neri G, Noonan J, Pierpont EI, Reinker K, Roberts AE, Shankar S, Sullivan J, Wolford M, Conger B, Santa Cruz M, Rauen KA (2014). Cardio-facio-cutaneous syndrome: clinical features, diagnosis, and management guidelines. Pediatrics.

[6] Noonan JA (2006). Noonan syndrome and related disorders: alterations in growth and puberty. Rev Endocr Metab Disord.

[7] Siegel DH, McKenzie J, Frieden IJ, Rauen KA (2011). Dermatological findings in 61 mutation-positive individuals with cardiofaciocutaneous syndrome. Br J Dermatol.

[8] Swan L, Coman D (2018). Ocular manifestations of a novel proximal 19p13.3 microdeletion. Case Rep Genet.

[9] Al-Kateb H, Hahn A, Gastier-Foster JM, Jeng L, McCandless SE, Curtis CA (2010). Molecular characterization of a novel, de novo, cryptic interstitial deletion on 19p13.3 in a child with a cutis aplasia and multiple congenital anomalies. Am J Med Genet A.

[10] de Smith AJ, van Haelst MM, Ellis RJ, Holder SE, Payne SJ, Hashim SK, Froguel P, Blakemore AI (2011). Chromosome 19p13.3 deletion in a patient with macrocephaly, obesity, mental retardation, and behavior problems. Am J Med Genet A.

[11] Nowaczyk MJ, Thompson BA, Zeesman S, Moog U, Sanchez-Lara PA, Magoulas PL, Falk RE, Hoover-Fong JE, Batista DA, Amudhavalli SM, White SM, Graham GE, Rauen KA (2014). Clin Genet.

[12] Corsello G, Giuffrè L (1991). Cardiofacio cutaneous syndrome: notes on clinical variability and natural history. Am J Med Genet.

[13] Rehman AU, Gul K, Morell RJ, Lee K, Ahmed ZM, Riazuddin S, Ali RA, Shahzad M, Jaleel A, Andrade PB, Khan SN, Khan S, Brewer CC, Ahmad W, Leal SM, Riazuddin S, Friedman TB (2011). Mutations of GIPC3 cause nonsyndromic hearing loss DFNB72 but not DFNB81 that also maps to chromosome 19p. Hum Genet.

[14] Duncan AMV, Anderson LL, Funk CD, Abramovitz M, Adam M (1995). Chromosomal localization of the human prostanoid receptor gene family. Genomics.

[15] Krauss M, Kukhtina V, Pechstein A, Haucke V (2006). Stimulation of phosphatidylinositol kinase type I-mediated phosphatidylinositol (4,5)-bisphosphate synthesis by AP-2 mu-cargo complexes. Proc Natl Acad Sci.

[16] Yang P, Chiang P-W, Weleber RG, Pennesi ME (2015). Autosomal dominant retinal dystrophy with electronegative waveform associated with a novel RAX2 mutation. JAMA Ophthal.

[17] Nardello R, Plicato G, Mangano GD, Gennaro E, Mangano S, Brighina F, Raieli V, Fontana A (2020). Two distinct phenotypes, hemiplegic migraine and episodic Ataxia type 2, caused by a novel common CACNA1A variant. BMC Neurol.

[18] Cefalu AB, Spina R, Noto D, Valenti V, Ingrassia V, Giammanco A, Panno MD, Ganci A, Barbagallo CM, Averna MR (2015). Novel CREB3L3 nonsense mutation in a family with dominant hypertriglyceridemia. Arterioscler Thromb Vasc Biol.

[19] Serra G, Memo L, Antona V, Corsello G, Favero V, Lago P, Giuffrè M (2021). Jacobsen syndrome and neonatal bleeding: report on two unrelated patients. Ital J Pediatr.

[20] Serra G, Antona V, Corsello G, Zara F, Piro E, Falsaperla R (2019). NF1 microdeletion syndrome: case report of two new patients. Ital J Pediatr.

[21] Corsello G, Antona V, Serra G, Zara F, Giambrone C, Lagalla L, Piccione M, Piro E (2018). Clinical and molecular characterization of 112 single-center patients with Neurofibromatosis type 1. Ital J Pediatr.

[22] Piro E, Serra G, Giuffrè M, Schierz IAM, Corsello G (2021). 2q13 microdeletion syndrome: report on a newborn with additional features expanding the phenotype. Clin Case Rep.

[23] Uludağ Alkaya D, Lissewski C, Yeşil G, Zenker M, Tüysüz B (2021). Expanding the clinical phenotype of RASopathies in 38 Turkish patients, including the rare LZTR1, RAF1, RIT1 variants, and large deletion in NF1. Am J Med Genet A.

[24] Aoki Y, Matsubara Y (2013). Ras/MAPK syndromes and childhood hemato-oncological diseases. Int J Hematol.

[25] Harms FL, Alawi M, Amor DJ, Tan TY, Cuturilo G, Lissewski C, Brinkmann J, Schanze D, Kutsche K, Zenker M (2018). The novel RAF1 mutation p.(Gly361Ala) located outside the kinase domain of the CR3 region in two patients with Noonan syndrome, including one with a rare brain tumor. Am J Med Genet A.

[26] Pride HB, Tollefson M, Silverman R. What's new in pediatric dermatology?: part I. diagnosis and pathogenesis. J Am Acad Dermatol 2013;68(6):885.e1–898. 10.1016/j.jaad.2013.03.001 23680204

[27] Serra G, Memo L, Coscia A, Giuffré M, Iuculano A, Lanna M, Valentini D, Contardi A, Filippeschi S, Frusca T, Mosca F, Ramenghi LA, Romano C, Scopinaro A, Villani A, Zampino G, Corsello G (2021). Their respective scientific societies and parents’ associations. Recommendations for neonatologists and pediatricians working in first level birthing centers on the first communication of genetic disease and malformation syndrome diagnosis: consensus issued by 6 Italian scientific societies and 4 parents' associations. Ital. J Pediatr.

